# Nationwide trends in paediatric pacemaker implantation in France, 2014–2024

**DOI:** 10.1093/europace/euag114

**Published:** 2026-05-19

**Authors:** Jacques Blacher, Laura Semenzato, Emmanuelle Dufour, Antoine Rachas, Mahmoud Zureik, Valérie Olié

**Affiliations:** Arterial Hypertension and Cardiovascular Prevention Department; Diagnosis and Therapeutic Center, Hôtel-Dieu, AP-HP, Université Paris Cité; Inserm U1153, Nutritional Epidemiology Research Team (EREN), Centre for Research in Epidemiology and StatisticS (CRESS), Paris, France; EPI-PHARE Scientific Interest Group in Epidemiology of Health Products from the French National Agency for the Safety of Medicines and Health Products and the French National Health Insurance, Saint Denis, France; EPI-PHARE Scientific Interest Group in Epidemiology of Health Products from the French National Agency for the Safety of Medicines and Health Products and the French National Health Insurance, Saint Denis, France; EPI-PHARE Scientific Interest Group in Epidemiology of Health Products from the French National Agency for the Safety of Medicines and Health Products and the French National Health Insurance, Saint Denis, France; EPI-PHARE Scientific Interest Group in Epidemiology of Health Products from the French National Agency for the Safety of Medicines and Health Products and the French National Health Insurance, Saint Denis, France; University Paris-Saclay, UVSQ, University Paris-Sud, Inserm, Anti-Infective Evasion and Pharmacoepidemiology Unit/Team, CESP, Montigny le Bretonneux 78180, France; EPI-PHARE Scientific Interest Group in Epidemiology of Health Products from the French National Agency for the Safety of Medicines and Health Products and the French National Health Insurance, Saint Denis, France

**Keywords:** pediatric, Pace maker, France

## Introduction

Pacemaker implantation in children requires even more careful consideration of the risk-benefit balance than in adult patients. Implanting a pacemaker in a child may be viewed as replacing one disease with another, with potential complications extending throughout the patient’s lifetime.^1^ Because this procedure remains relatively rare, the epidemiology of permanent pacemaker implantation in paediatric populations has been only sparsely described. We therefore analysed all pacemaker implantations performed in France between 2014 and 2024 in patients younger than 18 years to describe their characteristics and temporal trends.

## Methods

We used data from the French national hospital discharge database (PMSI), which records reimbursement data for inpatient care, including diagnoses, procedures, and implantable medical devices. These data were linked to the National Health Data System (SNDS), which contains pseudonymized health information for more than 99% of the French population. The pacemaker implantation was identified using procedure codes (from the French Common Classification of Medical Procedures [CCAM]) and reimbursable implantable pacemaker codes (from the List of Reimbursable Products and Services [LPP]). We identified all pacemaker implantations performed in children under 18 years between 2014 and 2024. Analysis of hospitalization diagnoses and procedures during the admission in which the pacemaker was implanted allowed us to determine the two most frequent paediatric aetiologies: high-grade congenital atrioventricular block and implantation following cardiac surgery for congenital heart disease.

## Results

**Figure 1:**
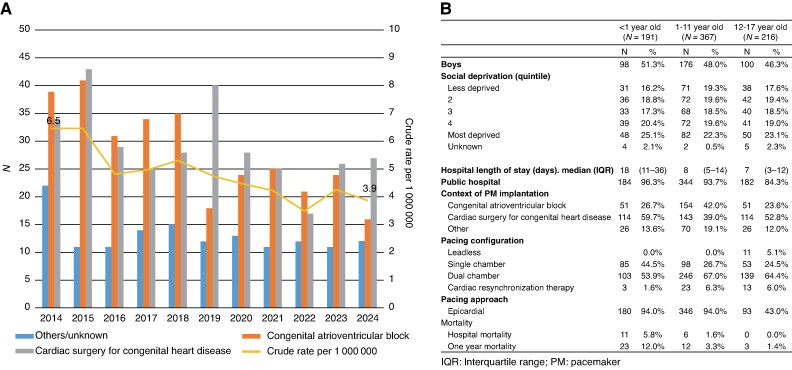
Paediatric pacemaker implantations in France 2014–2024. **A**: Temporal trends by pacemaker implantation indication. **B**: Characteristics of children implanted according to age group.

A total of 774 pacemaker implantations were identified in children younger than 18 years over the 11-year study period. We observed a progressive decline in the number of implantations, from 95 implantations in 2014 to 55 in 2024, with crude rates declining from 6.5 to 3.9 implantations per million population (*Figure 1A*).

The sex distribution was unbalanced across age categories (*Figure 1B*). Children living in the most socially deprived areas were overrepresented. The most frequent aetiologies were congenital atrioventricular block, accounting for 359 implantations (46%) and implantation following cardiac surgery for congenital heart disease (264 implantations, 34%). Implantation rate decreased over time for both indications. The vast majority of paediatric pacemaker implantations were performed in public hospitals. Single-chamber pacemakers were predominantly used. Leadless pacemakers accounted for 5,1% of implantations in children aged 12–17 years. Epicardial pacing was the most common approach in younger children, particularly among those undergoing cardiac surgery, whereas endocardial pacing became predominant from the age of 14 years onward. Hospital stays were prolonged, especially in the youngest age groups, with a median length of 18 days. In-hospital and 1-year mortality were higher in the youngest children (5.8 and 12%, respectively) than in the oldest.

## Discussion

In this nationwide cohort, we observed a declining implantation rate, predominance of congenital atrioventricular block and post-surgical indications, and age-dependent differences in device type and pacing approach.

The sex imbalance likely reflects the epidemiology of the two principal underlying conditions. Congenital atrioventricular block is more frequent in girls,^2^ whereas some severe congenital heart diseases are slightly more common in boys.^3^

The high proportion of single-chamber devices and the frequent use of epicardial pacing in younger children aligns with anatomical and surgical constraints. The shift toward endocardial pacing in adolescence reflects somatic growth and technical feasibility. Indeed, significant technical challenges may complicate lead implantation in small patients or those with abnormal venous anatomy^1,4,5^; unfortunately, neither weight, height, nor reason for epicardial or endocardial choice were available in our database. Furthermore, endocardial pacing in small children has to take into account risk of venous occlusion and later need for higher risk of endocardial leads extraction with long dwell times.^6^

Overall, pacemaker implantation decreased between 2014 and 2024, irrespective of aetiology. For high-grade atrioventricular block, accepted indications for implantation include symptomatic children, those with ventricular dysfunction, prolonged corrected QT interval, complex ventricular escape rhythms with wide QRS complexes, or ventricular pauses exceeding three times the basic cycle length.^4^ In contrast, over recent years, indications for implantation in asymptomatic children have become less stringent, with current recommendations advocating systematic implantation in children with a ventricular rate <50 bpm (previously <55 bpm).^5,7^

The second most frequent indication was pacemaker implantation following cardiac surgery for congenital heart disease repair, which also showed a progressive decline over time. In congenital heart disease, post-operative atrioventricular conduction block complicates 1% to 3% of cardiac operations.^8^ This decrease may be explained by three main factors. First, a reduction in the number of births of children with complex congenital heart disease, related to an increased frequency of therapeutic termination of pregnancy. Indeed, prenatal diagnostic accuracy has markedly improved since the 1980s, with detection rates reaching approximately 70% during the 2018–2020 period. In parallel, the proportion of therapeutic terminations of pregnancy for fetal anomalies increased between the 1980s and the 2020s, from 0.4% to 14%.^9^ Second, indications for permanent pacemaker implantation following postoperative atrioventricular block have also evolved. The most recent guidelines recommend definitive implantation 10 days after surgery (previously 7 days).^5^ Third, the reduction in the frequency of these implantations may also be related to the development of catheter interventions for correction of congenital heart disease, which better preserve the specialized conduction pathways, as well as to improvements in surgical techniques allowing improved preservation of the cardiac conduction system.^10^

In the absence of randomized trials, large-scale observational studies, and sufficiently comprehensive registries, indications for pacemaker implantation in children remain subject to debate (low level of evidence) in many indications in the most recent guidelines.^4^ Beyond the observed temporal trends, our study provides a rare nationwide descriptive overview of paediatric pacemaker implantation, including device type, implantation approach, socioeconomic distribution, and early mortality.

Although analysis of national databases has its own caveats with generally limited clinical data, we believe that the analysis of national multicentre medico-administrative data, comparison of practices, and centralized follow-up cohorts, including children who did and did not require pacemaker implantation (with comparative aetiologies of congenital heart block and congenital heart disease), could help provide more robust evidence and refine indications for pacemaker implantation in paediatric patients.

## Regulatory approval and ethical aspects

The National Health Data System (SNDS) is a set of strictly anonymous databases comprising all mandatory national health insurance reimbursement data, particularly data derived from the processing of healthcare claims (electronic or paper claims) and data from healthcare facilities (PMSI). The study was conducted in compliance with the French regulations on access and processing of personal data from the SNDS. EPI-PHARE has direct access to the SNDS from the permanent regulatory access of its constitutive bodies, the French National Agency for the Safety of Medicines (ANSM) and Health Products, and the French National Health Insurance (CNAM). Permanent access is given according to French Decree No. 2016-1871 of 26 December 2016 relating to the processing of personal data called the ‘National Health Data System’ 23 and French law articles Art. R. 1461-1324 and 1425. All requests in the database were made by duly authorized people, and this study was declared prior to its initiation on the EPI-PHARE registry of studies requiring the SNDS (T-2025-04-574).

## Data Availability

Jacques Blacher had full access to all the data in the study and takes responsibility for the integrity of the data and the accuracy of the data analysis.
